# Combustion Performance and Deposit Characteristics of Boron–Aluminum Composite Fuel in a Powder-Fueled Ramjet: A Ground Test Study

**DOI:** 10.3390/molecules30071503

**Published:** 2025-03-28

**Authors:** Zuodong Liang, Ming Jiang, Ronggang Wei, Hongyan Li, Shaoqing Hu, Kai Ma, Guiyang Xu, Wenjie Wang, Yanjing Yang

**Affiliations:** Xi’an Modern Chemistry Research Institute, Xi’an 710065, China

**Keywords:** powder-fueled ramjet, composite powder fuel, boron–aluminum, self-sustaining combustion, deposits analysis

## Abstract

Powder-fueled ramjets show great potential due to their unique advantages. How to further improve ramjet performance through methods such as fuel improvement is also an important focus. In this paper, a 14 km, Ma 3.0, ground test of a powder-fueled ramjet using boron–aluminum composite powder fuel (B–Al composite powder fuel) was conducted. The feasibility and combustion performance of B–Al composite powder fuel were verified. Under the condition of an air–fuel ratio of 19.39, the ramjet achieved independent self-sustaining combustion for 10 s, and the characteristic exhaust velocity efficiency ($\eta_{c}^{*}$) reached 81.84%. Through SEM-EDS, XRD, and XPS, this study systematically analyzed the surface morphology, composition, and chemical state of the wall deposits in the combustion chamber after the test. The combustion behavior of the B–Al composite powder fuel in the ramjet was clarified. The composite powder fuel could be converted into smaller and more combustion-favorable reaction basic units during the combustion process. However, the imbalance and unevenness of Al and B in the combustion reaction and the non-reaction or reaction termination of B particles remain significant issues. This study shows that B–Al composite powder fuel has a good application basis and potential, and provides experimental data support for the subsequent improvement and optimization of the B–Al composite powder fuel system.

## 1. Introduction

Powder-fueled ramjets have attracted extensive attention due to their unique advantages, such as high specific impulse [1], wide operating range [2], good stability [3], low storage requirements [4], and amphibious potential [5,6]. Since the concept of powder-fueled ramjets was born in the 1940s [7], numerous experimental studies [8,9] and theoretical studies [10,11] have shown that metal powder fuel, as a fuel with a high volumetric calorific value, has great potential.

At present, experimental research on powder-fueled ramjets mainly includes two systems: magnesium-based (Mg-based) fuel, and aluminum-based (Al-based) fuel. Since 2007, Shen et al. have conducted a series of theoretical studies on the thrust performance and internal flow field distribution of powder-fueled ramjets [12,13]. In 2009, their experiments demonstrated the self-sustaining stable combustion of a Mg-based powder fuel in ramjet combustion chambers [14]. Building on these studies, Hu achieved the goal of repeated start and stop in ground tests of an Mg-based powder-fueled ramjet, where the combustion efficiency reached a maximum of 72.8%. Experimental and theoretical performance discrepancies were noted, primarily due to the incomplete energy release [15]. Additionally, Li et al. modified the configuration of a powder-fueled ramjet, and through their ground tests, they adjusted the fuel supply parameters. As a result, the ramjet using Al metal powder fuel achieved 24 s of self-sustaining combustion, with a combustion efficiency of 73.05% [16].

In fact, the choice of high-energy metal elements that can be used in fuel is limited. Al has become the most widely used metal fuel and high-energy additive. In addition to the Al and Mg mentioned above, boron (B) with a higher volume calorific value has also been tried as a high-energy metal fuel. However, some characteristics of B make it difficult to fully realize its energy potential. One of the main problems of B is its slow and difficult burning [17]. Many studies have tried to solve this problem through various methods. One method is to add suitable catalysts to the B fuel. Catalysts like cerium-based compounds and others can reduce the reaction activation energy, thereby reducing the ignition delay time of the B particles [18]. On the other hand, some types of catalysts like ${\mathrm{MoO}}_{3}$ and others can improve the self-sustaining combustion characteristics of the B particles by destroying the oxide layer or providing an oxygen channel through the catalyst [19,20].

In addition, adding other metal fuels with good combustion characteristics, such as Al and Mg, to B fuel to improve its combustion characteristics is another a way to solve the problem of B combustion difficulties. These enhancements have been proven in fuel combustion experiments [21,22]. These experiments analyzed the intermediate products, employing in situ XRD and other methods. They determined the optimization of the reaction path of B fuel using metals such as Al and Mg. At the same time, these studies made it clear that adding other metal fuels with good combustion characteristics to B fuel, whether by mixing or constructing suitable compounds, can effectively shorten the ignition delay time of B fuel and extend the self-sustaining combustion time of the fuel [23,24,25].

In this paper, a ground test of a powder-fueled ramjet using B–Al composite powder fuel was conducted. The successful stable and self-sustaining combustion operation of the powder-fueled ramjet verified the feasibility and combustion performance of the B–Al composite powder fuel. The analysis of the deposits in the combustion chamber revealed the combustion behavior of the powder fuel during the actual working process. This study provides experimental insights into B–Al composite fuel combustion behavior, offering practical implications for powder-fueled ramjet design.

## 2. Results and Discussion

### 2.1. Combustion Performance

**Figure 1:**
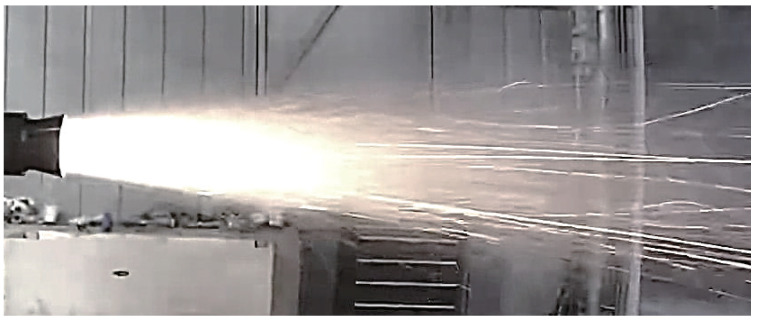
Flame of the powder-fueled ramjet during the experiment.

The experiment ran successfully as designed and expected. The powder-fueled ramjet completed ignition and a 10 s self-sustaining combustion-independent operation stage. Figure 1 shows a photo of the flame from the nozzle taken during the experiment.

The flame was generally bright white, with orange-yellow edges and a small amount of white smoke. The flame color was uniform and symmetric, and the edges were regular. Thus, the combustion in the combustion chamber could be considered smooth. Some bright white streaks were observed farther away from the nozzle exit. These streaks were considered large droplets or particles that had not been wholly reacted or cooled in the combustion chamber. Due to their smaller specific surface area, these large aggregates cooled more slowly, leading to prolonged combustion reactions or cooling processes. These bright white streaks were more likely to be Al droplets in reaction, $B_{2}O_{3}$ droplets, or ${\mathrm{Al}}_{2}O_{3}$ particles at high temperatures [26,27,28]. However, without appropriate temperature measurement methods or sampling analysis, it was impossible to identify and confirm the specific composition of these particles or droplets.

The main parameters obtained are presented in Table 1. These results were acquired using the measurement system and calculation methods mentioned earlier. The measured total pressure of the ram air was 0.40 MPa, and the total temperature was 606 K, which perfectly matches the design conditions. By measuring the static pressure within the gas generator, the gas flow rate during the operation of the gas generator was calculated. The fuel-rich gas flow rate was calculated to be 67 g/s, which also perfectly meets the design conditions.

The flow rate of the powder fuel calculated from the experiment was 87.70 g/s. This value showed a deviation of only 0.80% from the designed condition of 87.00 g/s. Consequently, the experimental air–fuel ratio of the powder-fueled ramjet was 19.39. The static pressure measurements at different axial positions in the combustion chamber indicated that the evolution of static pressure at various axial distances was almost identical. Within a range of 400 mm, the delay time of pressure evolution was less than 1 ms. Therefore, the measurement methods used in the experiment could not effectively distinguish the propagation of combustion signals. During the 10 s of self-sustaining combustion operation of the ramjet, the average static pressure in the combustion chamber was 0.2199 MPa. Based on these data, the performance of the powder-fueled ramjet and B–Al composite powder fuel could be calculated.

The combustion efficiency of the ramjet during operation could be evaluated using the characteristic exhaust velocity efficiency ${\eta_{c}}^{*}$, which was calculated using the following equation

(1) $${\eta_{c}}^{*}=\frac{c_{exp}^{*}}{c_{th}^{*}}$$

Here, $c_{exp}^{*}$ is the experimental characteristic exhaust velocity of the ramjet. $c_{th}^{*}$ is the theoretical characteristic exhaust velocity of the ramjet, representing its theoretical performance under complete combustion. This study calculated $c_{th}^{*}$ using thermodynamic methods based on local conditions by CEA [29,30]. The equation for $c_{exp}^{*}$ is

(2) $$c_{exp}^{*}=\frac{P_{c}A_{t}}{{\dot{m}}_{fc}+{\dot{m}}_{a}}$$

where $P_{c}$ is the static pressure of the combustion chamber, and $A_{t}$ is the throat area of the ramjet’s nozzle. ${\dot{m}}_{a}$ is the mass flow rate of ram air, while ${\dot{m}}_{fc}$ is defined to calculate the mass flow rate of the fuel and other components with fuel. The equation for ${\dot{m}}_{fc}$ is

(3) $${\dot{m}}_{fc}={\dot{m}}_{f}+{\dot{m}}_{g}+{\dot{m}}_{fg}$$

${\dot{m}}_{f},{\dot{m}}_{g}$ and ${\dot{m}}_{fg}$ are the mass flow rates of the powder fuel, fuel-rich gas, and fluidization gas, respectively. These components also contribute to the thrust of the powder-fueled ramjet.

The relevant performance metrics for the experiment could be calculated using the equations above. The results are shown in Table 2. The characteristic exhaust velocity efficiency of this experiment reached 81.84%, which was higher than the experimental studies shown in Table 3.

Overall, the experimental results demonstrate not only the feasibility and good performance of B–Al composite powder fuel, but also the advantages of the optimized powder-fueled ramjet configuration. This achievement also provides a new reference direction for the subsequent development and research of powder-fueled ramjets.

### 2.2. Deposit Analysis

In order to have a more detailed understanding of the combustion state of the B–Al composite powder fuel in the powder-fueled ramjet, this study conducted a series of analyses on the wall deposits in the combustion chamber after the ground test. This section will discuss the surface morphology, elemental composition, chemical state, and other information of two wall deposits located at the head (Sample 01) and the middle (Sample 02) of the combustion chamber. Sample 01 was located upstream of the ram air inlet, while Sample 02 was located downstream of the ram air inlet. The two samples represent deposits in the combustion chamber’s two typical reaction environments: the head environment upstream of the ram air inlet, and the mid-downstream environment after the ram air is supplemented. Thus, this study could comprehensively understand the state of deposits in the combustion chamber. To ensure the reliability of the sampling position, the sampling process selected deposits that were firmly attached to the wall, excluding any naturally peeled and movable parts.

In this study, the microstructure, elemental distribution, and chemical state of wall deposits in the ramjet combustion chamber were systematically studied using scanning electron microscopy combined with energy dispersive X-ray spectroscopy (SEM-EDS), X-ray diffraction (XRD), and X-ray photoelectron spectroscopy (XPS). A MIRA3 TESCAN model field emission scanning electron microscope (SEM) combined with an energy dispersive X-ray spectrometer (EDS) was used for morphological characterization. The sample preparation process glued the sample on a conductive adhesive. The probe for surface morphology characterization was a secondary electron detector (SE). The typical acceleration voltage was set to 20 kV, and the magnification was 50 kx. The EDS area scanning mode performed elemental mapping to resolve the surface composition heterogeneity. The study of crystalline phase composition used a Rigaku SmartLab model XRD instrument equipped with Cu Kα radiation (λ=1.5406 Å). Diffraction patterns were recorded in the 2θ range 5–90° with a step size of 0.02° and a scan rate of 5° $\min^{-1}$. Chemical state analysis was performed on a ThermoFisher ESCALAB Xi+ model XPS instrument equipped with a monochromatic Al Kα source (hν=1486.6 eV). Survey spectra (0–1350 eV) were acquired with a 1 eV step size, while high-resolution spectra used a 0.05 eV step size. XPS curves were charge compensated by calibrating the C 1s peak (accidental carbon at 284.8 eV), and spectral deconvolution was performed using Shirley background subtraction.

#### 2.2.1. SEM-EDS Analysis

**Figure 2:**
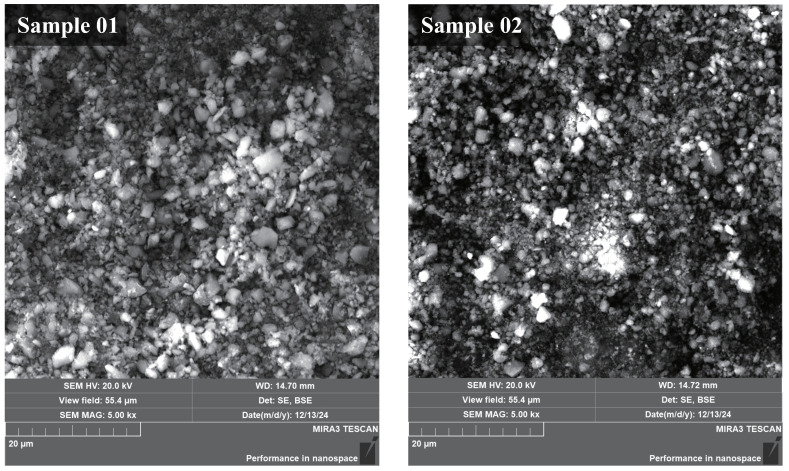
SEM images of the deposits.

From the SEM images in Figure 2, the surface morphology of Sample 01 and Sample 02 was similar overall. The surface particle size of the wall deposits was mostly distributed at 4 μm and below. This particle size is significantly smaller than the characteristic particle size of the fuel itself. This indicates that the actual combustion reaction was carried out in basic units of smaller size. There may be two factors behind this phenomenon. One factor is that the B fuel itself is agglomerated nano-sized B powder. These agglomerated B powders are significantly dispersed in the combustion chamber. Another factor is the introduction of AP into the B–Al composite fuel. AP has a micro-explosion effect during combustion. The micro-explosion effect will reduce the particle size of the particles and increase the specific surface area of the solid particles, thereby improving the combustion performance. Judging from the particle size distribution on the surface of the deposit, the aim of reducing the basic size of the reaction unit and thus improving the fuel combustion performance was achieved.

The EDS results showed the element distribution of the samples, and the results are shown in Table 4. From the difference between Sample 01 and Sample 02, Sample 01 had less B element and more O element, while N element and Al element accounted for a higher proportion. However, from the perspective of atomic ratio, it is not difficult to see that a considerable proportion of B element may not exist in the form of oxide, but in the form of a single substance. Assume that Al completely generates ${\mathrm{Al}}_{2}O_{3}$ and O only reacts with Al and B. In addition, assume that B only exhibits the two situations of generating $B_{2}O_{3}$ and not reacting. Then, the proportion of unreacted B in Sample 01 reached 75%. In Sample 02, this proportion reached 80%. This indicates that there was a large amount of unreacted B in the wall deposits. This may be because of the deposition of B that was not ignited, or it may be due to the deposition of B that had terminated combustion.

#### 2.2.2. XRD Analysis

**Figure 3:**
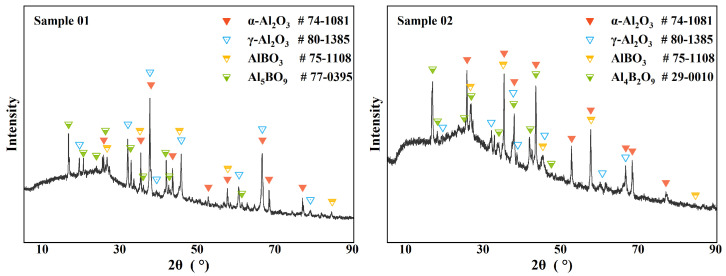
XRD survey spectrum of the deposits.

XRD can effectively analyze the composition of the crystalline phase materials in a sample. Figure 3 shows the XRD analysis results of the two samples. Previous studies have shown that the condensed phase products in the B-Al-N-O system usually include metal oxides, metal nitrides, and aluminoborates [21,22]. In this paper, the crystalline phase components obtained from the XRD analysis mainly included $\alpha -{\mathrm{Al}}_{2}O_{3}$, $\gamma -{\mathrm{Al}}_{2}O_{3}$, ${\mathrm{AlBO}}_{3}$, ${\mathrm{Al}}_{5}{\mathrm{BO}}_{9}$, and ${\mathrm{Al}}_{4}B_{2}O_{9}$. Overall, the composition of the crystalline components of the two samples was similar, but the signal strength of different compositions was different. In Sample 01, the $\gamma -{\mathrm{Al}}_{2}O_{3}$ signal was significantly stronger than the $\alpha -{\mathrm{Al}}_{2}O_{3}$ signal. In Sample 02, the more stable $\alpha -{\mathrm{Al}}_{2}O_{3}$ phase had a stronger signal. Considering the properties of the two alumina phases, the strong signal of $\gamma -{\mathrm{Al}}_{2}O_{3}$ indicated a larger temperature gradient at the head of the combustion chamber, which allowed the unstable crystal phase to be preserved.

As for the borate, the crystals present in Sample 01 were mainly ${\mathrm{Al}}_{5}{\mathrm{BO}}_{9}$, while the crystals present in Sample 02 were mainly ${\mathrm{Al}}_{4}B_{2}O_{9}$. Two pieces of information can be obtained from this. First, the ratio of ${\mathrm{Al}}_{2}O_{3}$ to $B_{2}O_{3}$ at the head and the middle of the combustion chamber was inconsistent, thus obtaining Al borates with different atomic ratios. Second, the wall temperature at the head and the middle of the combustion chamber was inconsistent, which affected the final stable crystals formed [34,35]. From the mutual conversion reaction, the wall temperature at the head of the combustion chamber was much higher than the wall temperature in the middle of the combustion chamber. On the other hand, ${\mathrm{AlBO}}_{3}$ was analyzed in both samples. The formation temperature of this crystal was lower, and the required ratio of ${\mathrm{Al}}_{2}O_{3}$ to $B_{2}O_{3}$ was also very different from the two Al borate crystals mentioned above. This shows that the reaction inside the combustion chamber was not uniform. This uneven reaction has two aspects. On the one hand, the enrichment area where B and Al react was not uniform, and on the other hand, the intensity of the reaction was uneven. In addition, the appearance of these aluminoborates indicates that in most wall environments, the amount of ${\mathrm{Al}}_{2}O_{3}$ was always greater than or much greater than $B_{2}O_{3}$. This difference in distribution density indicates that the oxidation reaction of Al was faster and had a higher reaction priority.

#### 2.2.3. XPS Analysis

**Figure 4:**
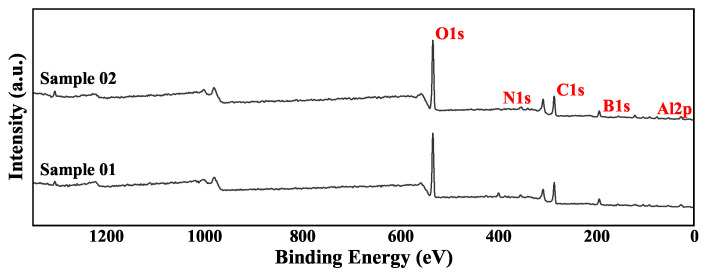
XPS survey spectrum of the deposits.

XPS is used to analyze the surface chemical state of a substance. Unlike XRD, it can analyze amorphous phase substances. But at the same time, XPS can only analyze shallow surface substances. The XPS total spectrum of the two samples is shown in Figure 4. The parts for fine spectrum scanning were mainly Al 2p and B 1s. This section analyzes the high-resolution spectrum of these two parts.

**Figure 5:**
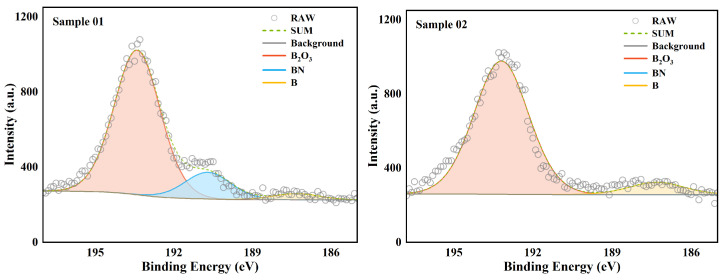
XPS high-resolution spectrum of B.

The high-resolution spectrum analysis of the 1s orbital of the B element is shown in Figure 5. The data of the compounds with different valence states are listed in detail in Table 5. Overall, the peak intensities of samples 01 and 02 were similar, and the main material composition of the surface was $B_{2}O_{3}$. The difference was that there was a clear presence of BN in Sample 01. In Sample 02, there was no BN. This indicates that there was a local oxygen-poor high-temperature environment at the head of the combustion chamber. This caused some B and nitrogen elements to combine to form BN substances. Since the combustion chamber temperature does not reach the melting point of BN [36], these BN stay in the wall deposits for a long time. In the middle of the combustion chamber, due to the supplementation of oxygen from the intake duct, the oxygen-poor environment no longer appears [37]. Therefore, there was no BN peak in the XPS analysis of Sample 02. This phenomenon is consistent with the analysis results of EDS. As for B elementary substance, there was a clear distribution in both Sample 01 and Sample 02. This is very different from the analysis results of EDS in terms of area ratio. This difference shows that the distribution of B elementary substance in the sediment was uneven. Compared with the very small amount of B elementary substance on the surface, the proportion of B elementary substance inside the wall sediment was very high. These B elementary substances inside the wall sediment were wrapped by other substances and had difficultly in contacting oxygen and heat. The internal B elementary substance basically had no chance to participate in the combustion reaction again. From the perspective of surface chemical state, B existed mainly in the form of complete oxide on the surface of the wall sediment. This shows that the oxidation reaction of B could continue under the condition of sufficient oxygen.

**Figure 6:**
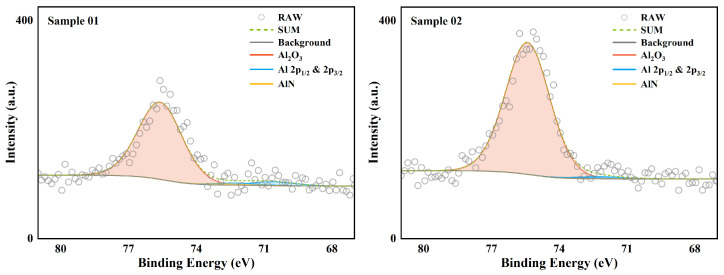
XPS high-resolution spectrum of Al.

Similarly, the high-resolution spectrum analysis of the 2p orbital of Al is shown in Figure 6. The data of compounds with different valence states are listed in detail in Table 6. Unlike element B, Al element existed in both samples in the form of ${\mathrm{Al}}_{2}O_{3}$ as the absolute main form. In addition, a very small amount of Al elementary substance was detected. This weak signal indicates the existence of a local thin ${\mathrm{Al}}_{2}O_{3}$ film. The Al nitride AlN almost completely disappeared in the XPS analysis. This shows that under the same environmental conditions, the oxidation reaction priority and reaction rate of Al were higher than those of B. Therefore, in an oxygen-poor environment, AlN will appear later than BN. This conclusion is consistent with the conclusion obtained by XRD.

Through the analysis of the morphology and components of the wall deposits in this section, the combustion behavior of the B–Al composite powder fuel in the ramjet was clarified. First, the composite fuel could form smaller basic reaction units with a larger specific surface area, more favorable for the combustion reaction during the combustion process. Second, there were significant differences in the reaction rates, reaction priorities, and reaction enrichment areas of Al and B, which require further optimization. Third, B could achieve complete oxidation reaction under the condition of sufficient oxygen, but at the same time, how to solve the problem of B particles not reacting or the reaction being terminated due to the encapsulation effect blocking oxygen contact remains an important issue. In addition, the differences and changes in the combustion environment in the head and middle of the combustion chamber require more in-depth and comprehensive research.

## 3. Powder Fuel

### 3.1. Fuel Selection

Metal fuels are widely recognized as superior energy carriers compared to hydrocarbon fuels in terms of their high energy density. Table 7 summarizes the properties of some typical high-energy metal elements, while Table 8 presents the properties of their main combustion oxides.

B has emerged as one of the most promising fuel components for powder-fueled ramjets, due to its exceptionally high volumetric calorific value (139.6 MJ/${\mathrm{dm}}^{3}$) and low biological toxicity [47]. However, unlike metal elements such as Al and Mg, the properties of B combustion products impose significant challenges in achieving reliable ignition and sustained combustion. These intrinsic limitations render B impractical as a single-component powder fuel for ramjet applications. As shown in Table 7, Al has a high volumetric calorific value (83.9 MJ/${\mathrm{dm}}^{3}$), which surpasses that of Mg (43.3 MJ/${\mathrm{dm}}^{3}$) and Lithium (Li, 22.3 MJ/${\mathrm{dm}}^{3}$), and ranks second only to B. In addition, previous studies have demonstrated sufficient success using Al as a single-component propellant in powder-fueled ramjets [16]. This combination of proven performance and favorable energy density makes Al a strategic compromise for composite fuel systems. Thus, this study selected Al as a partial substitute for B, constructing a B–Al composite powder fuel system. Although this reduces the energy and propulsion performance of the propellant, it may effectively improve the ignition and combustion performance of the powder fuel.

### 3.2. Composition and General Performance

**Figure 7:**
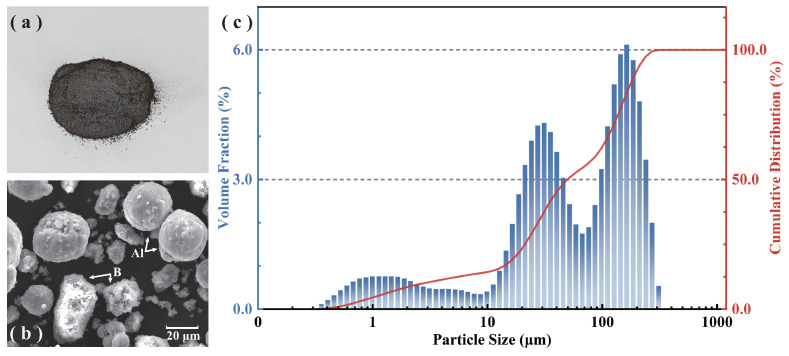
B–Al composite powder fuel (**a**) sample image, (**b**) SEM image, and (**c**) particle size distribution.

The composite powder fuel used in the study included spherical Al powder, micron-sized B powder agglomerated from nano B powder, Ammonium Perchlorate (AP), and an inert binder. The proportions are shown in Table 9. Figure 7a shows a sample picture of the composite powder fuel, and Figure 7b presents an SEM image of the composite powder fuel. The SEM image shows that the particles with good sphericity and smooth surfaces are Al particles, while the irregular and rough-surfaced particles are B particles. Due to the limitations of the agglomerated B powder preparation process, the particle size distribution of the B powder aggregates was relatively wide. $D_{50}$ refers to the median particle size of a powder material. Regarding particle distribution, the $D_{50}$ of the composite powder fuel was about 57μm, with the overall particle size distribution shown in Figure 7c. The $D_{50}$ of Al and agglomerated B were 29 and 200 μm, respectively. The composite powder can achieve a higher bulk density and volumetric calorific value with this ratio between the two diameters. And the presence of AP provides a micro-explosion effect for the powder particles, enabling larger particles to be transformed into smaller particles less than 10μm, which are more conducive to sustained combustion.

**Figure 8:**
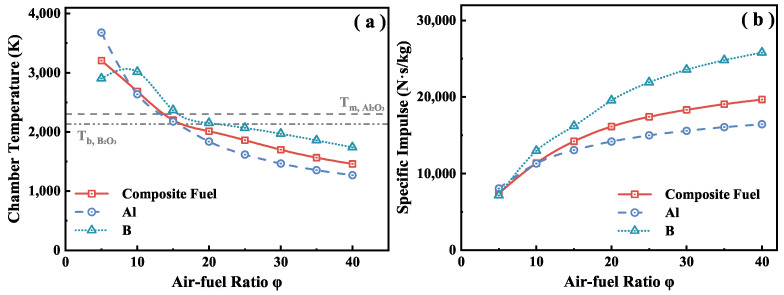
Relationship between chamber temperature (**a**), specific impulse (**b**), and air–fuel ratio. Flight height = 14 km, Ma 3.0. Results calculated by NASA CEA [29,30].

Figure 8 shows the specific impulse performance and combustion chamber temperature of B, Al, and B–Al composite fuel at different air–fuel ratios. The design point of the air–fuel ratio is mainly determined based on the specific impulse, and the deposition of condensate products also needs to be considered. Studies have shown that condensate products in the combustion chamber are prone to greater energy losses in liquid or gaseous form than in solid form, which is detrimental to the overall performance of a ramjet [11,16]. As shown in Figure 8a, the combustion chamber temperature is higher than the melting point of ${\mathrm{Al}}_{2}O_{3}$ (2303K) and the boiling point of $B_{2}O_{3}$ (2133K) when the air–fuel ratio is less than 19. When the air–fuel ratio is further increased, these gaseous or liquid products are converted into liquid or solid phases, due to decreased combustion chamber temperature. This phenomenon significantly helps reduce energy loss in the combustion chamber. Based on this characteristic, the air–fuel ratio in this study was selected as 19.48.

## 4. Experimental System

**Figure 9:**
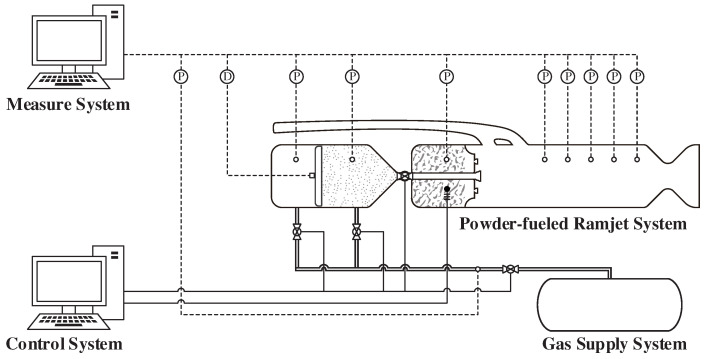
Schematic of the experiment system.

The experiment in this study was conducted on a ground-based ramjet test platform. The complete experimental system comprised a ram air simulation system, powder-fueled ramjet, control system, measure system, and high-pressure gas supply system. The schematic of the experiment system is presented in Figure 9. In this figure, “P” represents the pressure sensor, while “D” represents the displacement sensor. The symbol consisting of a solid circle with three horizontal bars represents the ignition device, which is governed by an independent isolated circuit.

### 4.1. Ramjet

**Figure 10:**
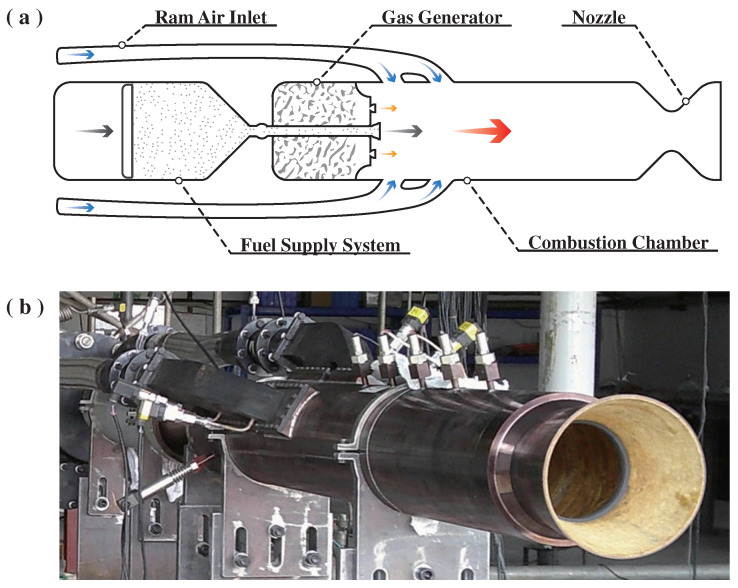
Powder-fueled ramjet system configuration (**a**) and prototype (**b**).

The powder-fueled ramjet constituted the core system of this experiment. As depicted in Figure 10a, the ramjet incorporates a ram air inlet, fuel supply system, gas generator, combustion chamber, and nozzle. The dual-side ram air inlet connects upstream to the ram air simulation system and downstream to the combustion chamber. The ram air simulation system provides the inlet with simulated air flow. Each inlet is split into two distinct flow channels, with differential mass flow rates and inflow angles. The forward ram air inlet channel provides the equivalent air–fuel ratio required for ignition, while the aft ram air inlet channel increases the combustion chamber’s air–fuel ratio, thereby improving the combustion efficiency and specific impulse performance [37]. A choked powder fuel supply system effectively mitigates combustion chamber back pressure effects on the stability of the fuel supply [55]. Critical components of this fuel supply system include the cylindrical chamber, sealed piston, fluidizing gas pipe, driving gas pipe, and fuel cut-off valve [56]. The piston divides the cylindrical chamber into two hermetically isolated compartments. As illustrated in Figure 10a, the compartment connected to the fluidizing gas pipe and fuel cut-off valve contains the powder fuel. During operation, the cut-off valve opens, while gas is introduced through separate pipes into distinct compartments. The resultant pressure differential drives piston displacement, propelling the powdered fuel through the cut-off valve into the combustion chamber. Adjusting the cut-off valve throat diameter regulates the supply mass flow rate. The pre-test confirmed that the powder fuel supply mass flow rate exhibited a maximum deviation of 5.0%, satisfying the experimental requirements.

In this study, the powder-fueled ramjet did not incorporate an ignition device within its combustion chamber. The requisite ignition energy was provided by sustained high-temperature fuel-rich gas from the gas generator. The propellant used in the gas generator was a B-rich oxygen-lean formulation, with an oxygen coefficient of approximately 0.127. Its chemical empirical formula can be expressed as $C_{15}H_{33}O_{12}B_{35}{\mathrm{Fe}}_{0.3}N_{2.4}{\mathrm{Cl}}_{2.6}K_{0.4}{\mathrm{Mg}}_{2.0}{\mathrm{Al}}_{0.6}$. During operation, the gas generator continuously discharges high-temperature, fuel-enriched gas containing substantial solid B particles into the combustion chamber. This B-enriched propellant configuration is recognized for effectively enhancing the specific impulse performance of powder-fueled ramjets. The combustion chamber is the primary structure for powder fuel combustion. To prolong the powdered fuel residence time and improve combustion efficiency, a swirl stabilizer is integrated at the head of the combustion chamber [16]. The ram air, fuel-rich gas, and powder fuel mix and combust within the combustion chamber, producing a high-temperature pressurized gas. This high-energy gas subsequently expands through the nozzle and exhausts, thereby generating thrust.

The operating range of a ramjet in subcombustion mode is usually within the flight height range of 10–15 km, with a flight velocity between 2.5 and 4.0 Mach. Much of the previous experimental research and flight tests were carried out within this operating range [16,57]. In this study, a flight altitude of 14 km and Ma 3.0 were selected as the design operating conditions of the powder-fueled ramjet, to meet the research requirements. The design conditions of the experiment are presented in Table 10. The design parameters of the powder-fueled ramjet are presented in Table 10, too. The combustion chamber was constructed with a 210 mm diameter and 1000 mm length. The nozzle was configured with a 128 mm throat diameter. Based on fuel characteristics, the air–fuel ratio was set to 19.48. A photo of the operational powder-fueled ramjet prototype is presented in Figure 10b.

### 4.2. Control and Measure

The control system was responsible for the actuating valves, ignition device, and other components within the experimental system. The measurement system operated synchronously with the experiment to monitor parameters such as pressure. A schematic of the control and measurement device configuration is shown in Figure 9. In this experiment, the primary measured data included the piston displacement in the fuel supply system and the static pressure in the combustion chamber of the powder-fueled ramjet. All measurement devices were sampled at a frequency of 1000 Hz.

The piston displacement in the fuel supply system was measured using a draw-wire displacement sensor (model: MOLINT MPS-XXS-600). This sensor has a measurement range of 0–600 mm, delivering a 4–20 mA current output signal. Its linearity accuracy is specified as 0.15% of full scale (FS), with a repeatability accuracy of 0.02% FS. The piston displacement data were utilized to calculate the powder fuel mass flow rate. The mass flow rate of powder fuel can be calculated following [56].

(4) $${\dot{m}}_{f}=\rho_{f}\pi r^{2}\cdot \frac{\Delta D_{p}}{\Delta t}$$

In this equation, $\rho_{f}$ is the bulk density of the powder fuel, and *r* is the radius of the cylindrical chamber. $\Delta D_{p}$ is the displacement distance of the piston within the operating time, Δt.

Five measurement points were set for static pressure monitoring in the combustion chambers to obtain the combustion state at different axial positions. The pressure taps were spaced at 100 mm intervals along the chamber axis, starting from 500 mm to 900 mm downstream along the head of the combustion. Static pressure data were acquired using diffused silicon pressure transmitters (model: MKYB3320) with a measurement range of 0–2.0 MPa. These transmitters exhibited a total accuracy of 0.10% FS. In addition, some other measure points were set in the measurement system to assess the subsystem operational status.

## 5. Experimental Procedure

The experimental procedure comprised three phases: preparation, test, and post-test. During the preparation phase, powdered fuel loading and system assembly were conducted. Concurrently, calibration procedures for the measurement system and functional verification of the control system were performed. The test phase defined the ignition timing of the gas generator as the temporal reference point (*t* = 0 s). At *t* = −5 s, the ram air simulation system was activated. Gas generator ignition occurred at *t* = 0 s, and the fuel supply system was initiated at *t* = +1 s. This triggered a 10 s autonomous operation phase of the powder-fueled ramjet under self-sustained combustion conditions. The fuel supply system terminated at *t* = +11 s. The gas generator underwent programmed shutdown at *t* = +13 s, marking the conclusion of the test. Post-test operations included purging and others. The measurement system remained operational throughout the entire experimental procedure.

## 6. Conclusions

In this study, B-containing powder fuel was introduced into a powder-fueled ramjet. A ground test of the B–Al powder-fueled ramjet was conducted. The combustion chamber wall deposits were analyzed to infer the combustion behavior of the fuel. The main conclusions are as follows:

- At an air–fuel ratio of 19.39, the powder-fueled ramjet achieved 10 s of independent, self-sustaining combustion, and B–Al powder fuel is feasible and effective in ramjets. The characteristic exhaust velocity efficiency $\eta_{c}^{*}$ reached 81.84%. Such an improvement shows that fuel formulation and ramjet configuration optimization are effective methods for improving combustion efficiency. At the same time, the performance shows that the B–Al composite powder fuel meets the requirements of subcombustion mode powder-fueled ramjets.
- With the analysis of combustion chamber wall deposits, the combustion behavior of B–Al composite powder fuel in the ramjet was clarified. The composite powder fuel could be converted into smaller and more combustion-friendly basic reaction units during the combustion process. However, the imbalance and unevenness of Al and B in the combustion reaction, as well as the non-reaction or reaction termination of B particles, remain important issues that need attention. The composition of such chamber deposits indicates that there is still much room for improvement in the fuel’s combustion efficiency. Subsequent research should combine the flow process in the combustion chamber to perform a more comprehensive analysis of the fuel’s combustion behavior. Based on this analysis, the fuel formula and fuel preparation method could be targeted for optimization.

It should be pointed out that although this study verified the feasibility and combustion characteristics of B–Al composite powder fuel in subcombustion mode ramjets, the current experimental condition in this paper was sufficiently unique that it cannot fully reflect the performance of B–Al composite powder fuel. In the future, the test conditions should be expanded to a broader range of flight heights and velocities, to evaluate the fuel performance comprehensively. At the same time, based on optimizing the fuel preparation process, the regulation mechanism of B–Al synergistic combustion with different particle size ratios and coating processes should be explored to further solve the problem of delayed B particle reaction and promote the development of powder-fueled ramjets towards a higher energy and wider range.

## Figures and Tables

**Table 1:** Measured parameters from the experiment.

Parameter	Value
Bulk density of powder fuel, $g/{\mathrm{cm}}^{3}$	1.21
Piston total displacement, mm	23.50
Powder fuel flow rate, g/s	87.70
Fuel-rich gas flow rate, g/s	67
Ram air flow rate, g/s	3000
Ram air total temperature, K	606
Ram air total pressure, MPa	0.40
Air–fuel ratio	19.39
Experiment time, s	10
Combustion chamber static pressure, MPa	0.2199

**Table 2:** Performance of the B–Al composite powder-fueled ramjet.

Parameter	Value
Experimental characteristic exhaust velocity $c_{exp}^{*}$, m/s	896.97
Theoretical characteristic exhaust velocity $c_{th}^{*}$, m/s	1096.00
characteristic exhaust velocity efficiency ${\eta_{c}}^{*}$, %	81.84

**Table 3:** Comparison of the combustion efficiency of the experiment in this study with the results of previous experimental studies with $P_{c}$ under 1.0 MPa.

No.	$P_{c}$	Fuel	ϕ	${\eta_{c}}^{*}$
01 [16]	0.34 MPa	Al	16.22	69.30%
02 [16]	0.31 MPa	Al	15.83	73.10%
03 [31]	0.59 MPa	AP/Al	12.15	46.90%
04 [31]	0.68 MPa	AP/Al	13.64	54.30%
05 [32]	0.77 MPa	AP/Al	21.46	68.70%
06 [33]	0.39 MPa	AP/Al	22.49	69.79%
07 [33]	0.38 MPa	AP/Al	21.76	65.03%
this paper	0.22 MPa	B/Al	19.39	81.84 %

**Table 4:** Atomic (%) composition of elements obtained from EDS.

Sample	B	C	N	O	Al
01	50.57	7.72	4.07	30.16	7.48
02	60.81	9.67	0.35	24.52	4.65

**Table 5:** Electron binding energy of B [38,39,40].

Parameter	$B_{2}O_{3}$	BN	B
Binding energy (eV)	193.40	190.70	187.30

**Table 6:** Electron binding energy of Al [41,42,43].

Parameter	${\mathrm{Al}}_{2}O_{3}$	$\mathrm{Al}\;2p_{1/2}$	$\mathrm{Al}\;2p_{3/2}$	AlN
Binding energy (eV)	75.60	72.72	72.26	70.60

**Table 7:** Properties of typical high-energy metal elements [44,45,46,47].

Element	Molar Mass	ρ	$T_{m}$	$T_{b}$	$Q_{m}$	$Q_{v}$	$\phi_{\mathrm{air},\mathrm{th}}$
	**(g/mol)**	**(g/cm^3^)**	**(K)**	**(K)**	**(MJ/kg)**	**(MJ/dm^3^)**	
Li	6.94	0.53	454	1617	42.1	22.3	4.95
B	10.81	2.37	2348	4273	58.9	139.6	9.53
Mg	24.31	1.74	922	1363	24.9	43.3	2.82
Al	26.98	2.70	933	2793	31.1	84.0	3.82

**Table 8:** Properties of main metal oxides from combustion [48,49,50,51,52,53].

Main Oxides	Molar Mass	ρ	$T_{m}$	$\Delta H_{\mathrm{fus}}$	$T_{b}$	$\Delta H_{\mathrm{vap}}$
	**(g/mol)**	**(g/cm^3^)**	**(K)**	**(kJ/mol)**	**(K)**	**(kJ/mol)**
${\mathrm{Li}}_{2}O$	29.88	2.01	1723	25	2736	${\text{-}\;}^{*}$
$B_{2}O_{3}$	69.63	2.46	723	22	2133	178
MgO	40.31	3.58	3098	77	3873	350
${\mathrm{Al}}_{2}O_{3}$	101.96	3.97	2303	109	3250	510

* ${\mathrm{Li}}_{2}O$ decomposes into gaseous Li and $O_{2}$ at high temperatures rather than being vaporized directly [54].

**Table 9:** Composition and proportion of B–Al composite powder fuel.

Ingredient	B	Al	AP	Inert Binder
Mass fraction (%)	42	50	4	4

**Table 10:** Design parameters of the powder-fueled ramjet.

Parameter	Value
Flight height, km	14
Flight Mach number	3.0
Ram air total temperature, K	606
Ram air total pressure, MPa	0.40
Ram air flow rate, g/s	3000
Powder fuel flow rate, g/s	87
Fuel-rich gas flow rate, g/s	67
Air–fuel ratio	19.48
Combustion chamber diameter, mm	210
Combustion chamber length, mm	1000
Nozzle throat diameter, mm	128

## Data Availability

Data will be made available on request.

## Abbreviations

The following abbreviations are used in this manuscript:

${\dot{m}}_{f}$	mass flow rate of powder fuel
$\rho_{f}$	bulk density of the powder fuel
*r*	radius of the fuel supply system’s cylindrical chamber
$\Delta D_{p}$	displacement distance of the fuel supply system’s piston
Δt	operating time of the fuel supply system
ρ	density
$T_{m}$	melting point
$T_{b}$	boiling point
$Q_{m}$	mass calorific value
$Q_{v}$	volumetric calorific value
$\phi_{\mathrm{air},\mathrm{th}}$	theoretical stoichiometric air–fuel ratio
ϕ	air–fuel ratio
$\Delta H_{\mathrm{fus}}$	enthalpy of fusion
$\Delta H_{\mathrm{vap}}$	enthalpy of vaporization
$D_{50}$	median particle size of the powder material
${\eta_{c}}^{*}$	ramjet’s characteristic exhaust velocity efficiency
$c_{exp}^{*}$	ramjet’s experimental characteristic exhaust velocity
$c_{th}^{*}$	ramjet’s theoretical characteristic exhaust velocity
$P_{c}$	static pressure of the ramjet’s combustion chamber
$A_{t}$	throat area of the ramjet’s nozzle
${\dot{m}}_{a}$	mass flow rate of ram air
${\dot{m}}_{fc}$	mass flow rate of the fuel and other components with fuel
${\dot{m}}_{g}$	mass flow rate of fuel-rich gas
${\dot{m}}_{fg}$	mass flow rate of fluidization gas
2θ	diffraction angle of XRD
